# A Bout of High-Intensity Interval Training (HIIT) in Children and Adolescents during Acute Cancer Treatment—A Pilot Feasibility Study

**DOI:** 10.3390/cancers14061468

**Published:** 2022-03-12

**Authors:** Sabine Kesting, Peter Weeber, Martin Schönfelder, Anja Pfluger, Henning Wackerhage, Irene von Luettichau

**Affiliations:** 1 Kinderklinik München Schwabing, Department of Pediatrics and Children’s Cancer Research Center, TUM School of Medicine, Technical University of Munich, 80804 Munich, Germany; peter.weeber@tum.de (P.W.); irene.teichert-vonluettichau@mri.tum.de (I.v.L.); 2 Chair of Preventive Pediatrics, Department of Sport and Health Sciences, Technical University of Munich, 80992 Munich, Germany; 3 Pediatric Oncology Network Bavaria, KIONET Bavaria, 91054 Erlangen, Germany; henning.wackerhage@tum.de; 4 Exercise Biology, Department of Sport and Health Sciences, Technical University of Munich, 80809 Munich, Germany; martin.schoenfelder@tum.de (M.S.); anja.pfluger@tum.de (A.P.)

**Keywords:** childhood cancer, high-intensity interval training, exercise intervention, adrenaline, lactate, heart rate, safety, feasibility

## Abstract

**Simple Summary:**

Exercise can counteract some of the adverse effects of cancer and its treatment. Epidemiological and mechanistic data suggest that exercise can influence cancer hallmarks, survival, and recurrence. Our pilot study aimed to assess the feasibility and safety of a single bout of high-intensity interval training (HIIT) in childhood cancer patients. The predefined feasibility criteria included recruitment rate, acceptability, practicability, and data acquisition. Very strict inclusion criteria and surveillance guaranteed safety, and no severe adverse events occurred. Our HIIT protocol is applicable only in a small number of childhood cancer patients. Blood lactate concentrations and heart rates significantly increased after HIIT, indicating that the patients achieved the targeted high exercise intensity. In conclusion, our preliminary data suggest that HIIT is safe and feasible in a small number of childhood cancer patients who do not suffer from severe side effects of treatment. Additional exercise protocols should be developed for patients with pronounced cancer-related impairments and health restrictions.

**Abstract:**

Low- and moderate-intensity exercise is safe and feasible during childhood cancer treatment. The feasibility of a bout of high-intensity interval training (HIIT) in this population has not been analyzed to date. Pediatric cancer patients aged between 6 and 18 years were selected based on clinical conditions to perform ten sets of 15 s HIIT (>90% of estimated maximal heart rate (HR_max_)) and 1 min active recovery on a bicycle ergometer within the first three chemotherapy courses. We assessed safety and feasibility criteria and the following parameters: perceived exertion rate, heart rate, and lactate and adrenaline concentrations. Out of 212 eligible patients, 11 patients aged 13.9 ± 3.6 years (*n* = 7 ♂) with lymphoma, leukemia, rhabdomyosarcoma, nephroblastoma, and synovial sarcoma completed the bout of HIIT without serious adverse events. During exercise, patients reached a BORG value maxima of 16 ± 1.2, and their heart rates rose from 78 ± 17 beats per minute (bpm) at rest to 178 ± 12 bpm after exercise (90 ± 6% estimated HR_max_). The power-to-weight ratio was 2 ± 0.5 W/kg (watt per kilogram). Blood lactate concentrations increased from 1.09 ± 0.50 mmol/L (millimole per liter) at rest to 5.05 ± 1.88 mmol/L post-exercise. Our preliminary data suggest that HIIT is applicable only in a small number of childhood cancer patients. Individually adapted exercise protocols for patients with multiple impairments are needed.

## 1. Introduction

Cancer is rare in children, and even though disease mortality is low in childhood, cancer is still the most common cause of death by disease in children throughout Europe [1]. According to the International Association of Cancer Registries, in Europe, 35.000 children and adolescents are diagnosed with cancer each year. Of these, 80% are disease free within 5 years after diagnosis [2]. Both adult and childhood cancer are primarily treated with chemotherapy, surgery, and/or radiation. In recent years, exercise has additionally been utilized in supportive cancer care. Exercise not only improves wellbeing and fitness, but it may also have direct anti-cancer effects. Because of these benefits, some cancer associations, such as the Clinical Oncology Society of Australia [3] and the American College of Sports Medicine, now recommend exercise for all cancer patients [4]. For childhood cancer, the German Network ActiveOncoKids recently published a guideline for physical activity promotion during and after treatment [5]. In adult cancer patients, exercise is safe and feasible; can improve physical functioning; increase quality of life; and reduce side effects, such as chemotherapy-induced toxicities and cancer fatigue [6,7]. In addition, high-intensity interval training (HIIT) has been rendered applicable in adult cancer patients [8], as well as in adult cancer survivors [9]. Several lines of evidence suggest that physical activity and exercise directly affect cancer, and this, in turn, may affect cancer risk, survival, and recurrence. A large meta-analysis pooling data from 1.44 million adults found leisure-time physical activity to lower the risk of developing 13 different adult cancer types [10]. Moreover, observational studies report improved survival and reduced recurrence in patients with breast [11], prostate [12], or colorectal cancer [13] who are more physically active. Serum obtained from healthy sedentary adults after nine weeks of high-intensity endurance cycling was found to reduce viability and proliferative capacity in tumor cells in vitro [14]. The amino acid glutamine supports growth in cells that have high energy demands and synthesize large amounts of proteins and nucleic acids. The reduction in serum glutamine by acute exercise has been shown to inhibit cancer cell growth [15].

In children, physical activity generally plays a vital role in physical and social development [16,17]. Recent studies have demonstrated that physical activity programs for children with cancer are safe and feasible [18,19] and that they contribute to their functional mobility [20]. Positive effects have been found for physical and cardiorespiratory fitness, muscle strength, body composition, flexibility, and health-related quality of life [21]. However, there is a lack of knowledge about whether exercise affects tumor cells directly in pediatric cancer patients and about the potential mechanisms [22,23].

HIIT is a time-efficient form of training that may improve VO_2max_ (maximal oxygen consumption) and provide health-related benefits within 10 min. These effects are similarly achieved by training for one hour at moderate intensity [24]. In addition, cancer-modulating hormones, such as catecholamines adrenaline and noradrenaline, increase at a more pronounced rate after high-intensity exercise [25] compared to after low-intensity exercise. Catecholamines have been linked to some anti-cancer effects of exercise, such as the accumulation of natural killer cells [26], an increase in antitumor immunity, and more CD8+ T cell tumor infiltration [27], as well as the activation of the Hippo tumor suppressor pathway [28]. It is unclear though if HIIT is safe and feasible in children with cancer and whether children are able and motivated to perform such exercise. The scientific community agrees that there is a need for age- and health-appropriate exercise prescriptions in the childhood cancer population.

The aims of our preliminary study were therefore to evaluate the safety and feasibility of a single bout of HIIT (10 × 15 s) in pediatric cancer patients. The outcome variables were chosen with respect to the following aspects: We focused on feasibility and safety criteria to ensure practicability and safe application of this intervention as required for follow-up studies. We observed physiological parameters to evaluate the responsiveness in this particular situation. Moreover, we analyzed the concentrations of lactate and adrenaline as objective parameters for exercise load. Additionally, adrenaline has been linked to anti-cancer effects in adults. If our protocol proves feasible and results in physiological responses, this will emphasize the need for mechanistic research and provide conditions for studies specifically focused on childhood cancer patients.

## 2. Materials and Methods

### 2.1. Study Design and Supervised HIIT Intervention

We conducted this cross-sectional monocentric study between March 2018 and April 2021 at the Department of Pediatrics and Children’s Cancer Research Center at the Technical University of Munich. Participation was voluntary, and informed written consent, including all information about study procedures, was signed by each participant, as well as by their legal guardian. All collected data were encoded (pseudonym) and in accordance with privacy policy standards. The Ethics Committee of the School of Medicine of the Technical University of Munich approved the study protocol (protocol code 535/17 S; 16 February 2018 and amendment 25 March 2019) and gave approval to the informed consent.

**Figure 1:**
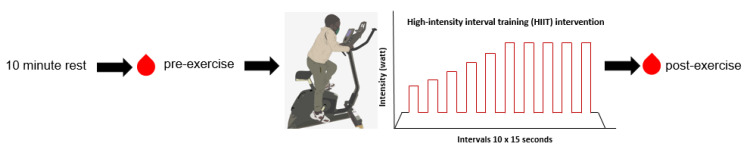
High-intensity interval training (HIIT) intervention protocol, including point of time for blood samples.

Participants performed a single supervised HIIT protocol with individual workloads on a bicycle ergometer as shown in Figure 1. To the best of our knowledge, no protocol for HIIT interventions in childhood cancer patients has been published to date. Regarding the feasibility character of our study and the vulnerability of our group of patients, we decided to use a protocol with 10 intervals of 15 s followed by 1 min of active recovery. We intended to reach an intensity of >90% of estimated HR_max_ (maximal heart rate) [29] by increasing the workload individually during the intervals. This protocol is based on the characteristics of HIIT interventions in children and adolescents summarized in the systematic review of Eddolls and colleagues [30] in combination with an expert discussion, and it was created with respect to the presumably impaired physical capacity of childhood cancer patients during acute treatment.

Following recruitment and prior to the intervention date, participants were informed again about the study procedures via telephone call. Participants were told not to exercise intensely the day before the intervention and to have a light breakfast, to avoid caffeine and high sugar-containing foods (e.g., chocolate and bananas), and to fast two hours before. These precautions were taken to avoid the potential influence of these foods on adrenaline levels according to the manufacturer’s specifications of the adrenaline measurement kit.

On the day of the intervention, participants came for a regular visit to the outpatient-clinic. First, participants underwent routine care procedures in the outpatient clinic, and the attending physician gave medical consent to perform the exercise intervention. Anthropometric data (height, weight) and vital parameters (heart rate, blood pressure) were collected. Second, after ten minutes of rest in a seated position, staff took the first blood sample via the central venous catheter following standardized clinical procedures. Third, the exercise physiologist adjusted the heart rate monitor and guided each participant through a low-intensity warm-up, mobilizing the joints for two minutes (see Section 2.3.3). Fourth, the bicycle ergometer was adjusted to the participants’ height. For participants shorter than 1.40 m, we used an electromagnetically braked children’s bicycle ergometer (Corival Pediatric, Lode, Groningen, The Netherlands), and for participants taller than 1.40 m, we used an adult ergometer with an induction brake (Kettler Ergometer Tour 300, Trisport AG, Hüneburg, Switzerland). Prior to the first interval, participants cycled for one minute at the lowest level of intensity (children ergometer: 0 watt, adult ergometer: 25 watt). The exercise physiologist announced the start of the HIIT, and participants were encouraged to cycle as fast as possible against the resistance, at a minimum rate of 60 rotations per minute (rpm). For the first interval, a low and individual workload was chosen with respect to the patients’ weight and height. Participants performed the intervention in a seated position without a face mask. Our protocol consisted of ten sets of 15 s HIIT (Figure 1). After each interval, participants cycled during an active recovery period of one minute with a low-intensity burden of 25 watt on the adult ergometer and 0 watt on the children’s ergometer. During active recovery, patients were told not to stop cycling but to continue pedaling. Immediately after each interval, the participants were asked to state their subjective rate of perceived exertion according to an adapted BORG scale (6–20), appropriate for children (Borg 1962, Figure 2). The next interval was adjusted according to the named number on the scale. During the intervention, we aimed to achieve a subjective exertion rate of 14–16 (yellow-red section on the scale), and participants were informed about this aim prior to the intervention. The load for each interval was individually increased in increments of 10–50 watts according to the participants’ feedback on the BORG scale, as long as the red section was not reached. Immediately after the last interval, participants laid down on the examination couch, and the second blood sample was taken within two to three minutes after finishing the last interval. Following the intervention, participants filled out the physical activity questionnaire ActiOn (see Section 2.3.4).

Vital parameters were measured prior to the intervention. Patients rested 10 min in a sitting position prior to the collection of blood samples. Processing of blood samples was conducted immediately.

### 2.2. Participants

We screened all newly diagnosed patients at our institution according to the following inclusion criteria: (1) diagnosed with pediatric cancer (new diagnosis or relapse), (2) aged between 6 and 18 years, (3) planned or implanted central venous catheter, (4) ability to follow study instructions in German, and (5) signed informed consent by patient and legal guardians and medical consent by the attending physician. The following exclusion criteria were applied: (1) medical contraindication regarding an intense exercise intervention (e.g., suspicious heart echocardiography, increasing pain during exercise, orthopedic impairments of the lower extremities) and parameters regarding an acute infection (temperature > 38 °C), blood counts below a certain level (platelet levels < 50.000 per μL, neutrophil count < 500 cells per μL, and hemoglobin < 8 g/dL) [31], and blood pressure values at rest according to age to ensure circulatory stability; (2) intellectual disability causing inability to follow study instructions; (3) inability to follow study instructions in German; (4) no signed informed consent; and (5) participation in another clinical exercise trial at the same time. Recruitment and intervention were conducted as early as possible following diagnosis and within the first three cycles of chemotherapy.

### 2.3. Procedures and Trial Endpoints

#### 2.3.1. Feasibility and Safety

We planned and conducted this project as a feasibility study referring to Thabane’s and colleagues’ review on pilot studies [32]. To define feasibility of our intervention, the following criteria were determined: recruitment, acceptance, practicability, and data acquisition. Recruitment is rated as feasible if at least 50% of eligible patients addressed agree with participation in the study. Acceptance is rated as given if at least 50% of participants finish the intervention. Practicability is defined as the realization of the intervention with respect to this specific cohort and the strict inclusion criteria within the estimated time investment of 6 h per participant on average (including preparations and data analysis). Data acquisition is rated as given if applicable data of all participants for analysis are collected (subdivided into subjective and objective data). Safety is defined as no serious adverse events and only adverse events of grade 1 (without consequences) occurring during the intervention [33]. This was also defined by the CTCAE (Common Terminology for Adverse Events) [34]. In order to minimize risks and to ensure safety during HIIT, we adhered closely to the following preventive guidelines:

- The attending physician gives medical consent regarding the capability to participate (see also Section 2.2 exclusion criteria) and appropriate blood values to perform HIIT;
- The intervention is conducted in a room within the outpatient clinic with medical supervision during and after the intervention;
- Low-intensity warm-up, including mobilization of the joints;
- Participants perform the intervention in a seated position to avoid tangling of the central venous catheter with the bike’s handlebar;
- Follow-up observation of every participant for 30 min after finishing the intervention;
- At least two individuals in the room during the intervention in case of an emergency;
- Close observation of the participant, including objective signs of exertion (e.g., flushed cheeks, paleness, intense breathing, quality of coordination and movement).

#### 2.3.2. Lactate and Adrenaline Concentrations

Blood samples were taken prior to and directly after the intervention within two to three minutes. From each whole blood sample, 20 µL was pipetted into 2.0 mL safelock microcentrifuge lactate tubes pre-filled with hemolytic solution (EKF Diagnostics, Magdeburg, Germany). Lactate samples were frozen for no longer than 14 days at −20 °C until measurement using a Biosen C-Line device (EKF Diagnostics, Magdeburg, Germany). Serum samples were established with 2.2 mL of whole blood collected in a 2.7 mL S-Monovette^®^ (Sarstedt, Nümbrecht, Germany). After 25 min of coagulation in an upright position, collection tubes were centrifuged for ten minutes at 2260 g and 20 °C. After centrifugation, serum was aliquoted by 300 µL into 1.5 mL Eppendorf^®^ safelock microcentrifuge tubes (Eppendorf Tubes, Hamburg, Germany), immediately snap-frozen on dry ice, and stored until analysis for maximum of one year at −80 °C. Plasma samples were established from 2.7 mL of whole blood collected in a 2.7 mL EDTA S-Monovette^®^ (Sarstedt, Nümbrecht, Germany). Plasma samples were centrifuged within five minutes after drawing and were processed using the same method used for the serum samples after centrifugation. Adrenaline concentrations were measured in plasma samples using the all-species adrenaline enzyme-linked immunosorbent assay (ELISA) kit (cat. No. LS-F5372, LifeSpan BioSciences Inc., Seattle, WA, USA) according to the manufacturer’s protocol. Plasma adrenaline concentrations were measured in pre- and post-exercise samples using a Tecan Sunrise plate reader and Magellan Software tool (Tecan, Männedorf, Switzerland). Adrenaline was measured in duplicate, and mean values were subjected to statistical analysis.

#### 2.3.3. Objective and Subjective Exhaustion Parameter

The rate of perceived exertion (modified BORG scale) is a common subjective parameter used in pediatric exercise oncology [35,36,37]. Heart rate was measured using a chest strap (Polar RS 800CX, Kempele, Finland) as an objective measurement of intensity at the moment of the intervention and of the level of the participants’ exhaustion. Additionally, the exercise physiologist observed the participants closely for the usual visible signs of exertion (e.g., flushed cheeks, paleness, intense breathing, quality of coordination, and movement). The retrospective evaluation of exertion was measured by the level of lactate in the blood samples collected prior to and following the intervention.

#### 2.3.4. Physical Activity Pre-Diagnosis

We assessed physical activity with the pre-diagnosis version of the ActiOn questionnaire to compare the patients’ level of physical activity to that of healthy children and adolescents. This tool was recently developed by exercise physiologists with profound expertise in pediatric exercise oncology from the German Network ActiveOncoKids for childhood cancer patients and survivors [38]. It is based on validated physical activity questionnaires for children and adolescents and retrospectively assesses the estimated physical activity level before diagnosis. Validation of this questionnaire is pending. Questions about the number of days with at least 60 min of physical activity during a regular week and the number of days with moderate-to-vigorous physical activity are included. These questions reflect the 2020 recommendations of the World Health Organization for children and adolescents [39]. Reference data from the German Health Interview and Examination Survey for Children and Adolescents (KiGGS Wave 2, *n* = 6532 girls and 6449 boys between 2014–2017; Finger et al., 2018 [40]) were used for comparison with our data.

### 2.4. Statistical Analysis

All aspects and results regarding feasibility and safety are provided descriptively. Physiological endpoints were analyzed with inferential statistics. To test the pre/post differences between the average values of two data sets of repeated measures, we used a two-tailed paired *t*-test. Normality was proven by the Shapiro–Wilk Test. Significant difference was determined by *p* < 0.05. Statistical analysis was performed using SigmaPlot v13 software tool and illustrated by PRISM 9.2.0 tool (GraphPad, San Diego, CA, USA). Statistical evaluation was performed in consultation with the Institute of Medical Informatics, Statistics, and Epidemiology of the Technical University of Munich.

## 3. Results

The purposes of the present study were to evaluate the feasibility and safety of a bout of HIIT in a sample of childhood cancer patients, and to document the physiological response. Only 11 (5%) out of 212 eligible childhood cancer patients could be recruited. A total of 195 patients did not meet the inclusion criteria. The response of a single set of ten high-intensity interval bouts on outcomes of heart rate, blood lactate, rate of perceived exertion, and blood adrenaline concentration was analyzed in the participants.

### 3.1. Patient Recruitment and Characteristics

**Figure 3:**
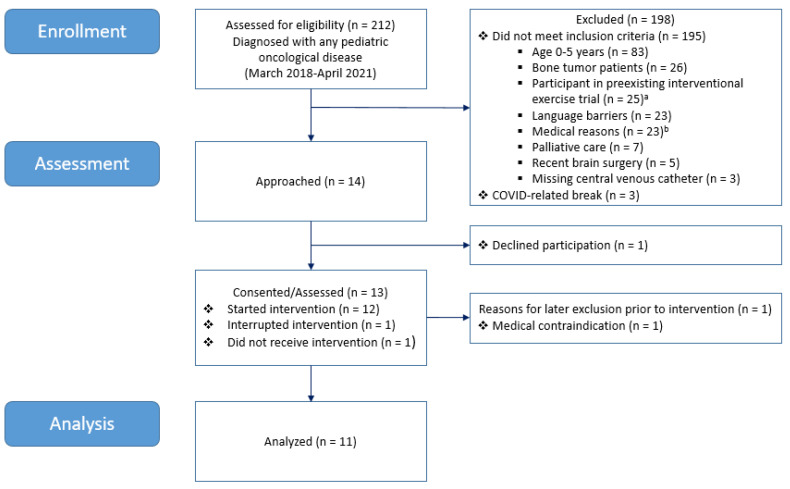
Flowchart of recruitment and study participation. *n*, number. ^a^ pre-existing exercise intervention study (ClinicalTrials.gov Identifier NCT03934060). ^b^ medical reasons for exclusion: severely reduced capacity (*n* = 13), treatment at intensive care unit (*n* = 3), osteoporotic vertebral compression fractures (*n* = 3), inability to sit/walk (*n* = 2), ventilated (*n* = 1), and severe comorbidity (*n* = 1).

Out of the 212 newly diagnosed and screened patients with a pediatric malignancy, 14 patients were included in our study within a recruitment period of three years. The majority (*n* = 195) did not meet the strict inclusion criteria that were applied for safety aspects. A huge number of 83 patients were aged zero–five years, and HIIT intervention was not applicable due to body height, suitable bicycle ergometers, and compliance. Patients with bone tumors located at the lower (*n* = 20) or upper extremity (*n* = 6) had to be excluded due to a prohibition of weight bearing of the affected limb and the risk of a pathologic fracture. Another 25 patients already participated in a pre-existing randomized controlled exercise intervention study during the whole period of acute treatment (ClinicalTrials.gov Identifier NCT03934060), and they were not included in this study due to good scientific practice. In 23 patients, language barriers hampered the procedures of recruitment and the following of instructions during the intervention. Another 23 patients were excluded due to medical reasons according to the attending physician (i.e., severely reduced capacity (*n* = 13), treatment at intensive care unit (*n* = 3), osteoporotic vertebral compression fractures (*n* = 3), inability to sit/walk (*n* = 2), ventilated (*n* = 1), and severe comorbidity (*n* = 1)). A burdensome palliative situation and recently conducted brain surgery led to the exclusion of seven and five patients, respectively, and intense exercise was not possible. Three patients did not receive a central venous catheter, and due to ethical reasons, peripheral blood collection for study purposes only was not approved by the Ethics Committee. Another three eligible patients could not be included due to the restriction of study procedures, including intense physical activity, during the COVID-19 pandemic. One patient declined participation prior to the intervention due to personal reasons. One recruited participant did not receive the intervention due to an infection. Another one started the intervention but discontinued after the first interval due to treatment-related nausea and was not able to continue. This patient suffered from pre-existing treatment-related nausea during hospital stays but, after physician approval, had agreed to perform the intervention despite this problem. A total of 11 (5%) participants completed the intervention. Figure 3 shows a flowchart of the recruitment and study participation.

The eleven participants were 13.9 ± 3.4 years old and had been diagnosed with different types of childhood malignancies (lymphoma, leukemia, rhabdomyosarcoma, nephroblastoma, and synovial sarcoma). A total of eleven participants finished the intervention and were analyzed. Table 1 provides further details about the participants’ medical and anthropometrical characteristics. Ten participants performed the HIIT on the adult ergometer and one participant (7 years, 1.25 m) used the children’s bicycle ergometer.

### 3.2. Endpoints

#### 3.2.1. Feasibility and Safety

Criteria for safety and feasibility were defined prior to the start of the study (Section 2.3.1). Table 2 summarizes the feasibility criteria and provides information about the fulfillment rate.

No serious adverse events and no adverse events with consequences occurred during the intervention [34]. One participant had to stop after the first interval due to pre-existing nausea in association with chemotherapy. Another participant had slight nose bleeding after the ninth interval, but this was stated to be a regular occurrence, even before cancer diagnosis, and they finished the intervention.

#### 3.2.2. Physical Parameters

**Figure 4:**
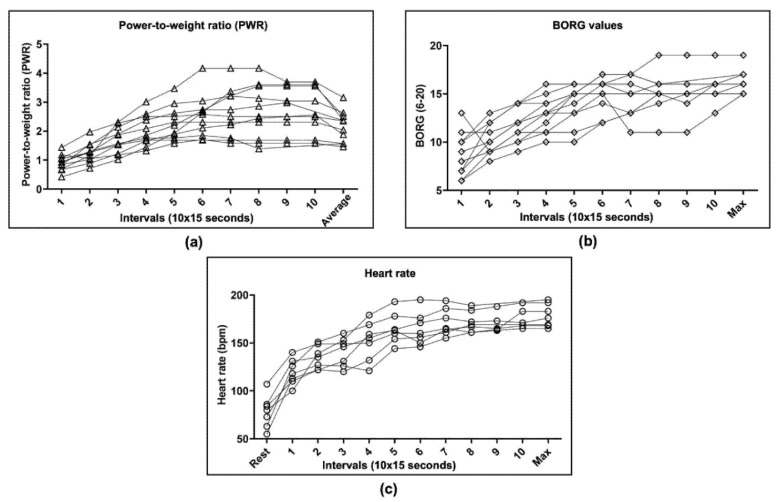
High-intensity interval training (HIIT) was performed with 11 childhood cancer patients after completing a two-minute warm-up. Between each interval, there was one minute of active recovery, during which the patients stated their estimated exertion level. (**a**) Power-to-weight ratio indicates the ratio of body weight to power output for ten intervals for all eleven childhood cancer patients. Average values are presented, excluding active recovery periods, as the mean of the 10 intervals per patient, which range from 1.46 to 3.16 W/kg. The maximum power levels reached range between 65 watts and 300 watts. (**b**) Patients provided information on their exertion level based on the BORG scale ranging from 6 to 20. After warm-up, BORG values were stated to be between 6 and 7, indicating a very, very low perceived exertion level. Maximum BORG values range from 15 to 19, indicating the perceived exertion level to be high and very high, respectively. (**c**) Heart rate, evaluated in only seven of eleven patients due to technical difficulties with the chest strap, increased significantly during exercise from 78 ± 17 bpm at rest to a maximum of 178 ± 12 bpm using a paired *t*-test (*p* < 0.0001). *n*, number; W, watt; kg, kilogram; PWR, power-to-weight ratio; bpm, beats per minute; max, maximal.

**Figure 5:**
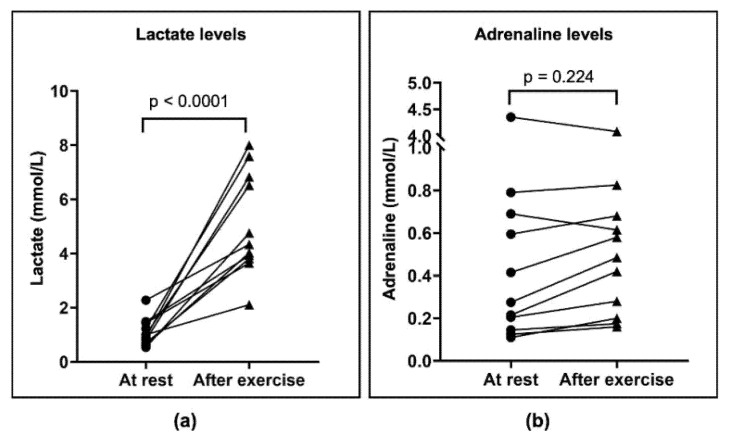
Lactate and plasma adrenaline concentrations were measured in blood samples obtained prior to and directly after exercise intervention. (**a**) There was a significant increase in lactate concentrations when comparing resting (1.09 ± 0.50 mmol/L) and post-exercise values (5.05 ± 1.88 mmol/L) using a paired *t*-test (*p* < 0.0001). (**b**) There was no significant change in adrenaline concentrations when comparing resting and post-exercise values, despite adrenaline concentrations showing high variability within the tested group. Low adrenaline concentrations of 0.2 nmol/L (nanomole per liter) are in line with available data in healthy children [42]. mmol/L, millimole per liter; nmol/L, nanomole per liter.

To characterize how much the bout of intense exercise affected the cardiovascular system, metabolism, and the cancer modulator adrenaline, heart rate was measured during the intervention, and blood lactate and adrenaline were measured at rest within three minutes post-exercise. Figure 4a,b show the continuous increase in power and perceived exertion in all analyzed patients (*n* = 11). Mean heart rate increased from 78 ± 17 bpm at rest to a HR_max_ of 178 ± 12 bpm post-exercise (Figure 4c) in *n* = 7/11 patients. This corresponds to 90% of the estimated HR_max_, as calculated using the Tanaka equation (HR_max_ = 208 − 0.7 × age), in children and adolescents [29,41]. In four patients, the measurement of heart rate was impaired due to technical difficulties with the chest strap. After exercise, blood lactate reached a maximum concentration of 5.05 ± 1.88 mmol/L (Figure 5a). Adrenaline concentration did not change from rest to post-exercise (Figure 5b).

#### 3.2.3. Physical Activity Pre-Diagnosis

Participants reported 60 min of moderate-to-vigorous physical activity in a regular week pre-diagnosis on 3.5 ± 2.5 days (median: 3 days, range: 0–7 days). Two participants met the WHO recommendations of 60 min of physical activity every day, and one was active for 60 min on six days in a normal week. These data are in accordance with the healthy reference population of the KiGGS study’s Wave 2, with 22.4% of girls and 29.4% of boys achieving the recommendations [40]. Five patients attended sport club activities regularly before diagnosis, eight engaged in leisure-time exercise activities, and all participated in physical education at school.

## 4. Discussion

The first major finding of this pilot study suggests that our HIIT protocol is not suitable for the majority of children during the first weeks of cancer treatment. We conclude this since only 14 of the 212 childhood cancer patients could be recruited for the study due to the restrictive inclusion criteria. Of these, 11 patients were able to finish a bout of HIIT. The second major finding is that the exercise intervention is feasible and safe for patients who suffer from only mild treatment side effects. The remaining 11 patients who completed a bout of HIIT demonstrate this finding: their heart rates increased to 178 ± 12 bpm and their blood lactate concentrations to 5.05 ± 1.88 mmol/L. Surprisingly, adrenaline did not change significantly but varied greatly inter-individually.

Between March 2018 and April 2021, a total of 212 children were hospitalized, but recruitment was challenging. The COVID-19 pandemic further hampered recruitment due to strict hygienic measures and a restriction of study procedures involving intense physical activity. Because of these measures, a large proportion of newly diagnosed patients under the age of six years were excluded from the intervention. Typically, all patients older than two years of age are offered participation in an individually adapted physical activity program. Our HIIT protocol is feasible with a very small number of physically fit childhood cancer patients; however, it cannot be applied as a general approach for this group. We observed no serious adverse events with consequences in our study. Only experienced staff attended to the patients during the study while adhering to specific safety prevention strategies [43] in order to minimize risk. Excluding one, all participants performed the intervention on the adult bicycle ergometer. One patient was analyzed on the children’s ergometer due to body height. This seven-year-old child was able and motivated to perform our HIIT protocol. However, the overall workload and recovery load are not comparable to the adult-sized ergometer. In general, exercise interventions are known to be feasible and safe during and after childhood cancer treatment [19,21], but research and data regarding HIIT during treatment have, to the best of our knowledge, not been published to date.

In addition to the careful selection of study participants for our HIIT intervention, the setting must also be considered. Recruitment within the first three cycles of chemotherapy treatment is challenging due to the short time frame and known treatment-related side effects (e.g., nausea, fever, infections, and mucositis). Taking preventive safety strategies (e.g., blood count, and cycles of chemotherapy) into account, patients’ and physicians’ approval to participate, and the insertion of a central venous catheter to take blood samples, timing is central to the success of the intervention. The demonstrated feasibility of an early HIIT intervention will allow for the measurement of potential tumor-influencing parameters early in the course of disease in future studies.

Our HIIT protocol caused an increased heart rate in the participating patients, which is in accordance with published data in healthy children and adolescents [44]. To the best of our knowledge, there are no existing data about adrenaline or lactate concentrations in childhood cancer patients following exercise. Surprisingly, the blood concentration of adrenaline did not increase when comparing it at rest to post-exercise. This finding is in contrast to published data in adults [25] and healthy children. We do not assume chemotherapeutical interference in adrenaline accumulation in childhood cancer patients. Although we know that the two- to three-minute half-life period of adrenaline is rather short, we managed to take blood samples within this time frame, while also adhering to hygienic guidelines. It is possible that the overall workload throughout our protocol could be too low to produce an increased blood concentration of adrenaline. Since adrenaline concentration can increase during HIIT in relation to mean exercise intensity [45], an extension of our HIIT protocol might have resulted in increased adrenaline levels.

As expected, we found a significant increase in blood lactate in the immediate post-exercise period, which validates the subjectively perceived exertion levels stated by the patients. There was high variability in power values (watt), which can be explained by the heterogeneity of our test subjects regarding age and fitness level. The power-to-weight ratios show comparable burdens throughout our studied group. Low power values (watt) also led to an increase in lactate, which shows that the individual watt adaption was successful in achieving high intensity. Consequently, we have planned a follow-up study to observe one group of physically fit childhood cancer patients performing a HIIT intervention with more intervals compared to one group of less physically fit childhood cancer patients performing a less intensive protocol. Our aim is to define a minimum feasible exercise burden that leads to a physiological response on a molecular level for the majority of childhood cancer patients.

The questionnaire data show that most children and adolescents in our study did not meet the WHO physical activity recommendations of at least 60 min of mostly aerobic physical activity prior to diagnosis. However, they were similarly physically active in comparison to their healthy peers [40]. The study participants could participate in a single bout of HIIT because they only experienced minor treatment side effects at the time of the intervention.

## 5. Conclusions and Perspective

The main finding of this study is that our HIIT protocol is safe and feasible in a small and physically fit group of childhood cancer patients. However, given that only about 5% of the eligible children and adolescents with cancer were capable of participating in this intervention due to our strict inclusion criteria, our HIIT protocol seems to be unsuitable for inclusion in the treatment and supportive care regimen for childhood patients with cancer on a regular basis. Our findings contribute to a better understanding of the applicability of HIIT in the field of pediatric exercise oncology. There is growing interest in mechanistic research on the effect of exercise; however, the focus of this research remains on adult cancer patients. We aim to expand our studies with the goal of determining the effects of exercise at the molecular level in childhood cancer patients. This represents an essential new research perspective beyond current research programs on soft but important outcome parameters (e.g., quality of life and physical fitness). Specifically, we propose the following key aspects for future research in this field:

- The adaption of exercise tests specifically for childhood cancer patients is needed to define standardized workloads for training protocols.
- The development of individually adapted exercise protocols (low-intensity exercise training) for patients with multiple impairments and health restrictions due to their underlying disease and cancer treatment.
- The application of repeated HIIT interventions and analysis of physiological parameters during the entire course of chemotherapy treatment.
- The initiation of multicenter studies to generate a greater sample size and increase informative value.
- The analysis of metabolite concentration changes in exercise-conditioned serum to detect relevant exercise and cancer-related metabolites in children.

## Figures and Tables

**Figure 2:**
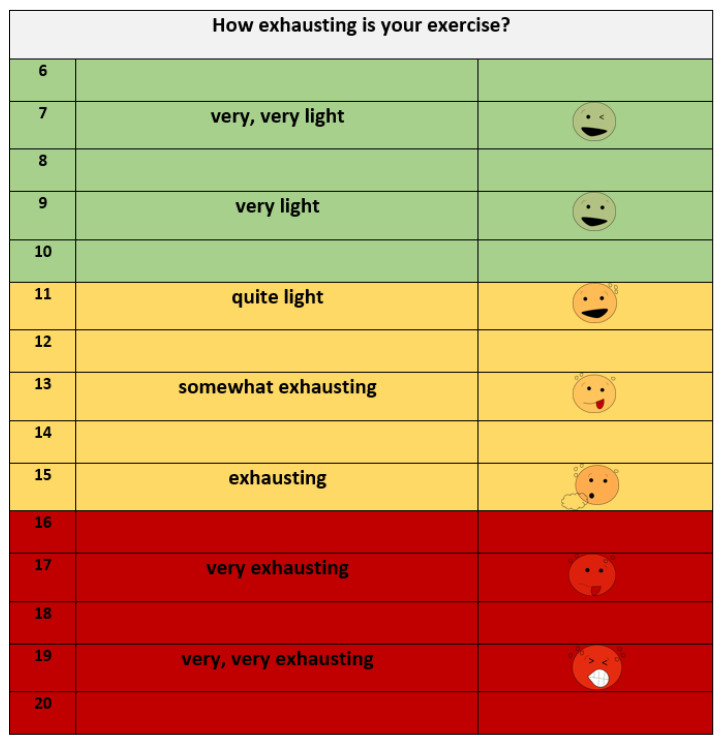
Scale for rating of perceived exertion (RPE scale, modified version of the BORG scale) (Borg 1962). The scale shows a range from 6 to 20. Participants were asked to estimate their subjectively perceived rate of exertion. For children and adolescents, the scale was modified by adding faces that visually reflect the level of exertion.

**Table 1:** Participant characteristics.

Characteristics	Study Group (Analyzed) *n* = 11
Age (mean ± SD, median, [range], years)	
At diagnosis	13.8 ± 3.4, 15 (7–18)
At intervention	13.9 ± 3.6, 15 (7–18)
Age group (*n*)	
Children (6–11 years) ^a^	3
Adolescents (12–17 years)	6
Young adults (>17 years)	2
Sex (% male)	
Male	64
Female	36
Time since Diagnosis (mean ± SD, median [range], days)	
	55 ± 11, 54 (34–74)
Cancer type	
Leukemia	2
Lymphoma ^b^	6
Other solid tumor ^c^	3
Anticancer treatment received until examination	
Chemotherapy	12
Radiotherapy	0
Surgery ^d^	0
Last application of chemotherapy before the intervention (days) ^e^	9 ± 6 (2–20)
Anthropometrical variables (mean ± SD, median [range])	
Body weight (kg)	61.6 ± 20.2, 59.0 (21.6–90.0)
BMI (kg/m^2^)	20.7 ± 4.1, 20.6 (13.8–27.7)
Body surface (m^2^)	1.69 ± 0.37, 1.72 (0.85–2.18)
Physiological parameters (at rest) (mean ± SD, median [range])	
Heart rate (bpm)	78 ± 13, 78 (55–107)
Blood pressure systolic (mmHg)	117 ± 11, 111 (104–135)
Blood pressure diastolic (mmHg)	80 ± 9, 80 (68–97)
Physical activity pre-diagnosis ^f^ (mean ± SD, days)	
Days with physical activity ≥ 60 min per day	4 ± 3, 3 (0–7)
Days with moderate-to-vigorous intensity	4 ± 3, 3 (0–7)

*n*, number; SD, standard deviation; kg, kilogram; BMI, body mass index; m^2^, square meters; bpm, beats per minute; mmHg, millimeters of mercury; min, minute. ^a^ only one child at the age of 7 years with a body height of 1.25 m performed the HIIT on the children’s bicycle ergometer. ^b^ non-Hodgkin lymphoma and Morbus Hodgkin; *n* = 1 early relapse of Morbus Hodgkin. ^c^ rhabdomyosarcoma, nephroblastoma, synovial sarcoma. ^d^ indicates a surgical intervention other than implantation of a central catheter or biopsy for diagnostic reasons. ^e^ if cardiotoxic chemotherapeutical treatment was applied, period before HIIT was at least 7 days. ^f^ days with ≥ 60 min of any physical activity per week (normal week) prior to diagnosis and days with moderate-to-vigorous physical activity (normal week) prior to diagnosis.

**Table 2:** Feasibility criteria and rate of fulfillment.

Feasibility Criteria	Objective
Recruitment	>50% of addressed participants
Acceptance	>50% finish the intervention (83%; 10/12) *
Practicability	Realizable for a specific group of participants with respect to strict inclusion criteria and an average time investment of 6 h per participant as estimated, and participants achieve the intended level of intensity (subjectively via BORG scale and objectively via lactate concentrations)
Data acquisition	Applicable data of all participants
	Subjective data:
	Physical activity questionnaire (100%; 11/11)
	Rate of perceived exertion (BORG scale) (100%; 11/11)
	Objective data:
	Heart rate (64%; 7/11)
	Blood samples for lactate and adrenaline concentrations (100%; 11/11)

* One participant interrupted after the first interval due to pre-existing treatment-related nausea and therefore could not be included in the final analysis; one participant interrupted after the eighth interval due to muscular tiredness of the legs and was included in the final analysis.

## Data Availability

The data presented in this study are available on request from the corresponding author. The data are not publicly available due to privacy or ethical restrictions.
